# Helminthes in Feral Raccoon (*Procyon lotor*) as an Alien Species in Iran

**Published:** 2020

**Authors:** Meysam SHARIFDINI, Omar M. AMIN, Keyhan ASHRAFI, Nader KARAMZADEH, Iraj MOBEDI, Behnaz RAHMATI, Zahra HESARI

**Affiliations:** 1. Department of Medical Parasitology and Mycology, School of Medicine, Guilan University of Medical Sciences, Rasht, Iran; 2. Institute of Parasitic of Diseases, Scottsdale, AZ, USA; 3. Department of the Environment, Guilan Provincial Office, Rasht, Iran; 4. Department of Medical Parasitology and Mycology, School of Public Health, Tehran University of Medical Sciences, Tehran, Iran; 5. Department of Pharmaceutics, School of Pharmacy, Guilan University of Medical Sciences, Rasht, Iran

**Keywords:** Prevalence, Raccoons, Helminthes, Iran

## Abstract

**Background:**

The raccoon, *Procyon lotor* Linn. (Procyonidae) is native to North and Central America but has been introduced in several European and Asian countries including Japan, Germany and Iran. Objective of this study was to determine frequency of gastrointestinal and tissue helminthes from feral raccoons in Iran.

**Methods:**

During 2015–2017, 30 feral raccoons including 12 males and 18 females were collected from Guilan Province, northern Iran (the only region in Iran where raccoons are found). The gastrointestinal tracts and tissues such as lung, liver and muscles were examined for presence of helminthes.

**Results:**

Twenty raccoons (66.7%) were found infected with five intestinal helminth species. The prevalence of infection with *Strongyloides procyonis* Little, 1966 (Nematoda) was 63.3%, *Plagiorchis koreanus* Ogata, 1938 (Trematoda) (13.3%), *Centrorhynchus* sp. Lühe, 1911 (Acanthocephala) (10.0%), *Camerostrongylus didelphis* Wolfgang, 1951 (Nematoda) (3.3%), and *Spirocerca lupi* Rudolphi, 1809 (Nematoda) (3.3%). No larvae or adult worms were found in other tissues of the examined raccoons.

**Conclusion:**

Most of the raccoons were infected with *S. procyonis*. The public health importance of zoonotic parasites transmittable through raccoons, the rapid control and decrease of raccoon populations and their distribution in Iran are also discussed.

## Introduction

The raccoon, *Procyon lotor* Linn. is a medium-sized carnivore native to North and Central America. In the mid-20 ^th^ century, raccoons were introduced into Europe, Caucasia, and Japan as a result of deliberate or accidental introductions (1–4). The raccoons were released in some regions of former the Soviet Union between 1936 and 1958 for their valuable fur (5). The raccoon was first reported in northern Iran in 1991, near the Iran-Azerbaijan border, where it most likely had migrated from Azerbaijan (6). Since then, raccoons were observed more often in north of Iran, especially in Talesh and Astara regions (6, 7).

Raccoons serve as reservoir hosts for wide variety of zoonotic helminthic parasites species such as *Baylisascaris procyonis* McClure, 1933, *Strongyloides procyonis* Little, 1966, *Dirofilaria tenuis* Chandler, 1942, among others (8–10). *B. procyonis* can cause ocular or neural larva migrants in humans. Most diagnosed human cases have been in children and were severe or fatal (11). In spite of the increasing population and wider distribution of raccoons in Guilan Province (6), there is no study of the occurrence of helminth parasites in raccoon in Iran or even in Caucasia.

The current study reports the prevalence of gastrointestinal and tissue helminthes of feral raccoons in Guilan Province, northern Iran to assess their potential role in the zoonosis of human helminthiasis.

## Material and Method

### Study area

**Fig. 1:**
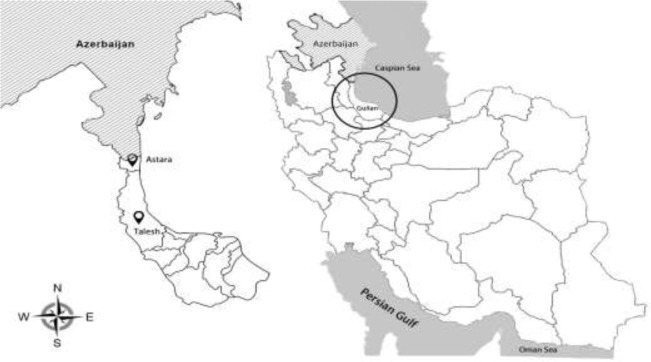
Map of Iran showing geographical location of Guilan Province and the study area, Astara and Talesh districts, in near the Iran–Azerbaijan border (7)

Guilan Province is located on southwest of the Caspian Sea and in the north of Iran. It has a surface area of 14,711 km ^2^ (between 36°36' and 38°27'N, and between 48°43' to 50°34'E) (Fig. 1) (6). The province has a humid subtropical climate with mean annual rainfall of 1359 mm (12). Current study area is limited to the western regions of Guilan Province including Astara and Talesh districts because reports of raccoon presence in Iran are limited to these areas (6).

### Sample Collection

Thirty feral raccoons were collected using trapping, hunting and road kills between Aug 2015 and May 2017. The sex and age group of each raccoon were recorded. Based on the life stage, the raccoons were categorized by the veterinarian as puppies or adults. Blood samples were taken from the animals' hearts that hunted or trapped (27 animals). Subcutaneous tissues were observed macroscopically for the presence of adult filarial worms. Necropsy was performed and internal organs including gastrointestinal tracts, heart, kidneys, lungs, liver, and muscles of the carcasses were removed in the field station. All the samples were preserved in 70% alcohol and transferred to the laboratory for further parasitological examinations.

### Parasitological Examinations

In the laboratory, small and large intestines were macroscopically screened for parasites by longitudinal dissection and the mucosa was scraped. The contents of the intestines were then washed using tap water runs through a sieve at 850 μm and 53 μm mesh and observed by stereomicroscope for the detection and recovery of helminths. Nematodes and acanthocephalans were cleared in lactophenol wet mounts and trematodes were stained using carmine alum. Every organ including esophagus, stomach, heart, kidneys, lungs, liver and bladder was examined separately under stereomicroscope. Thin sections of raccoon diaphragm and skeletal muscles were examined for larvae of *Trichinella* sp. Railliet, 1895 by trichinoscopy. Thin and thick smears were prepared for each blood sample and stained with 10% Giemsa for the presence of microfilaria. Species identification of the helminths was determined according to the morphological characteristics by valid key references (13, 14).

### Data analysis

Statistical tests including Chi-square (X2) and Fisher’s exact tests were used. Statistical analyses were performed using SPSS software version 18 (Chicago, IL, USA).

### Ethical approval

The protocol of this study was approved by the Ethics Committee of Gums University of Medical Sciences, Iran (Ref. No. IR.GUMS.REC.1394.185).

## Results

Twelve of the 30 raccoons examined (40%) were males and 18 (60%) were females. Twelve (40%) of the raccoons were puppies and the remaining 18 (60%) were adults. Twenty raccoons (66.7%) were found infected with at least one intestinal helminth species. There was a significant increase in the frequency of infection with all helminths identified in the puppies as compared to the adult raccoons (*P=*0.024). Infection rate was somewhat higher in male raccoons (83.3%) than in females (55.6%), but this difference was not significant (*P*=0.23) (Table 1).

**Table 1:** Frequency of helminthic parasites in feral raccoons from Guilan province, northern Iran according to the sex and age groups

***Helminth species***	***Raccoon age class***		***Raccoon sex***		***Total (N=30) No. (%)***
	***Pup (N=12) No. (%)***	***Adult (N=18) No. (%)***	***Male (N=12) No. (%)***	***Female (N=1.8) No. %***	
*Strongyloides procyonis*	11 (91.6)	8 (44.4)	9 (75)	10 (55.5)	19 (63.3)
*Plagiorchis koreanus*	3 (25)	1 (5.5)	2 (16.6)	2 (11.1)	4 (13.3%)
*Centrorhynchus* spp.	0 (0)	3 (16.6)	2 (16.6)	1 (5.5)	3 (10.0%)
*Camerostrongylus didelphis*	1 (8.3)	0 (0)	1 (8.3)	0 (0)	1(3.3%)
*Spirocerca lupi* larva	0 (0)	1 (5.5)	0 (0)	1 (5.5)	1(3.3%)

**Fig. 2:**
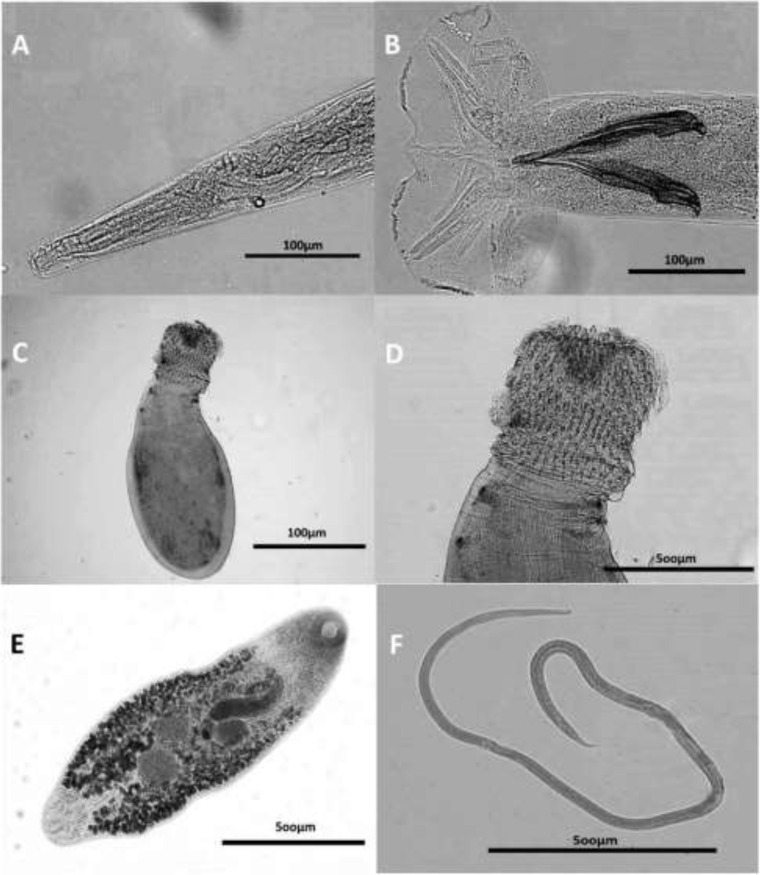
Light microscope view of the worms isolated from raccoons in Iran. Anterior end (A) and copulatory bursa (B) of *Camerostrongylus didelphis*. Proboscis (C) and general view (D) of *Centrorhynchus* spp. General view of *Plagiorchis koreanus* (E) and *Strongyloides procyonis* (F)

Helminthes identified comprised 3 species of nematodes, 1 species of trematode, and 1 acanthocephalan species. The most prevalent helminthic parasite in raccoons was *S. procyonis* (Fig. 2) (19 hosts, 63.3%). The rate of infectivity with other species was as follows: *Plagiorchis koreanus* (Fig. 2) (4, 13.3%), *Centrorhynchus* spp. (Fig. 2) (3, 10.0%), *Camerostrongylus didelphis* (Fig. 2) (1, 3.3%) and *Spirocerca lupi* larva (1, 3.3%).

The prevalence of infection with *S. procyonis* in puppies was significantly higher than in adults (*P*=0.018) but no statistically significant difference was found between *S. procyonis* infection and gender (*P*=0.44). For other species, there was no significant difference between sex and age groups with infection. No larvae or adult worms were found in the other organs of examined raccoons such as esophagus, stomach, heart, kidneys, lungs, liver, bladder, or subcutaneous tissue and muscles. Moreover, no microfilaria was found in peripheral blood smears.

Prevalence of infection with one, two, three species of helminthes was 43.3%, 20% and 3.3%, respectively. Co-infection is shown in Table 2. No statistical difference was found with age group or sex in co-infections.

**Table 2:** The co-infections of parasitic helminthes of feral raccoons from Guilan province, northern Iran

***Raccoons (n=30)***	***Co-infection of helminth parasites***	***No. (%)***
Double	*Strongyloides procyonis* + *Camerostrongylus didelphis*	1(3.33)
	*Strongyloides procyonis + Plagiorchis koreanus*	3 (10)
	*Strongyloides procyonis + Centrorhynchus spp.*	1(3.33)
	*Strongyloides procyonis + Spirocerca lupi* larva	1(3.33)
Triple	*Strongyloides procyonis* + *Plagiorchis koreanus* + *Centrorhynchus* spp.	1(3.33)

## Discussion

During the last century, raccoons were introduced to several European and Asian countries such as Iran as an alien invasive species (3). To date, raccoons have only spread in the western regions of Guilan Province in Iran. Raccoons contribute to the transmission of many zoonotic groups of helminthes to other wildlife and humans (9). Species introduced into a new environment often lose their own parasites during the course of establishment (15, 16) but may also acquire new parasites in the new regions.

In this study, the overall prevalence of intestinal helminths was 66.7%. A similar study in western Poland reported a relatively higher prevalence of 83.6% (17).

*B. procyonis* is readily transmissible to humans and appears to have the highest public health importance among raccoon parasites (18). This worm was not detected in the present study but studies involving larger sample size are needed for a complete evaluation. *B. procyonis* was also not recovered in feral raccoons in Japan (10, 19, 20).

In the present study, *S. procyonis* was the most frequent helminth parasite recovered from 63.3% of raccoons in Iran. The species was described for the first time in raccoons in the United States (21, 22) and was found in 25.5% of raccoons introduced in Japan (10). It was also identified in 11% of raccoons in western Poland using fecal sample examinations (23). In most other studies, *S. procyonis* was not found in the gastrointestinal tracts of raccoons (9, 17, 19, 24–28). In our study, puppies were considerably more frequently infected with *S. procyonis* (91.6%) than adults (44.4%). This was probably affected by vertical transmission of *S. procyonis* larvae, especially through the transmammary route. This is a common pathway in the life cycle of several species of *Strongyloides*, including *S. westeri* Ihle, 1917 in horses*, S. papillosus* Wedl, 1856 in cattle*, S. fulleborni* von Linstow, 1905 in human, *S. stercoralis* (Bavay, 1876) Stiles & Hassall, 1902 in dogs, *S. ransomi* Schwartz & Alicata, 1930 in swine, and *S. ratti* Sandground, 1925 and *S. venezuelensis* Brumpt, 1934 in rats (29). On the other hand, the animals may develop an immune response to parasite infections with increasing age.

In our study, *P. koreanus* had a prevalence rate of 13.3%. Puppies were more frequently infected than adults (25% vs. 5.55%). The difference was, however, not statistically significant. *P. muris* Tanabe, 1922 was found in 0.2% of raccoons in Japan (10). *P. vespertilionis parorchis* Macy, 1960 was reported in 2.4% of raccoons in British Columbia, Canada (27)*.*

In our study, 10.0% of raccoons were infected with *Centrorhynchus* spp. Species of the *Centrorhynchus* were also reported from raccoons in Japan (5.7%) (10). Species of this genus primarily infect birds but are occasionally reported from mammals and reptiles (30). They have also been reported from stray dogs (31), long-legged buzzard (32), and common buzzard (33) in Iran. This is probably due to infection of the introduced raccoons by the worm in environment of Iran.

*Spirocerca lupi* has a relatively wide range of definitive hosts that includes canids (dogs, jackals, coyotes, foxes, and wolves) as well as wild felids (23). This species was rarely found in ermine, *Mustela ermine* Linnaeus, 1758, and in the European polecat, *Mustela putorius* Linnaeus, 1758, as definitive hosts (34). Moreover, *S. lupi* was reported at relatively high prevalence (8.8%) in raccoons from Warta Mouth National Park (Poland) using fecal sample examination (23). Coprophagous (dung) beetles are the intermediate hosts of this worm (35). In this study, *S. lupi* larvae were detected from intestine of a raccoon. It was probably an accidental parasite due to high proportion of coprophagous beetles in the diet.

To the best of our knowledge, ours is the first report of the natural infection of a raccoon with *C. didelphis* in the world. Wolfgeng first reported and described *C. didelphis* in the small intestine of opossum in Trinidad (36). The introduction of infected raccoons from the American continent to Iran needs to be determined.

## Conclusion

Our study demonstrated the prevalence of helminth parasites in feral raccoons in the study area. The most prevalent parasite, *S. procyonis*, is known to have caused creeping eruption and infection of a healthy human volunteer experimentally infected (21, 22, 37). The public health importance of *S. procyonis* and other zoonotic parasites transmittable though raccoons appear to be related to the rapid control and decrease of population and distribution of raccoons in Iran. The prevalence and diversity of raccoon parasites in Iran appear to be lower than those reported from other countries.
